# Prognostic value of right ventricular dyssynchrony in adults with repaired tetralogy of Fallot

**DOI:** 10.1136/openhrt-2023-002583

**Published:** 2024-01-19

**Authors:** Andrea Papa, Clement Nussbaumer, Eleni Goulouti, Fabienne Schwitz, Kerstin Wustmann, Daniel Tobler, Matthias Greutmann, Markus Schwerzmann

**Affiliations:** 1 Center for Congenital Heart Disease, Department of Cardiology, Inselspital, University of Bern, Bern, Switzerland; 2 University Heart Center, University Hospital Basel, Basel, Switzerland; 3 Department of Congenital Heart Defects and Pediatric Cardiology, German Heart Centre Munich, Technical University Munich, Munich, Germany; 4 Adult Congenital Heart Disease Program, University Heart Center, University Hospital Zurich, Zurich, Switzerland

**Keywords:** Tetralogy of Fallot, Echocardiography, Diagnostic Imaging

## Abstract

**Objective:**

Residual sequelae after surgical repair of tetralogy of Fallot (rTOF) affect clinical outcome. We investigated the prognostic impact of right ventricular (RV) dyssynchrony in adults with rTOF years after the surgical repair.

**Methods:**

Patients from the Swiss Adult Congenital HEart disease Registry were included. NT-proBNP levels, echocardiography, exercise testing and MRI data were collected. An offline strain analysis to quantify RV-ventricular and interventricular dyssynchrony was performed. The standard deviation of the time-to-peak shortening (TTP) of six RV segments defined the RV Dyssynchrony Index (RVDI). Maximal difference of TTP between RV and left ventricular segments defined the interventricular shortening delay (IVSD). Predictors of a composite adverse event (arrhythmias, hospitalisation for heart failure and death) were identified by multivariate Cox regression analysis. Their median values were used to create a risk score.

**Results:**

Out of 285 included patients (mean age 34±14 years), 33 patients (12%) experienced an adverse event during a mean follow-up of 48±21 months. No correlation was found between RVDI, IVSD and clinical events. NT-proBNP, right atrial area and peak heart rate were independent predictors of outcomes. After 4 years-follow-up, no adverse events occurred in patients at low risk (score=0 points), while an adverse event occurred in 62% of patients at high risk (score=3 points, p<0.001).

**Conclusion:**

In our cohort of adults with rTOF, surrogates of RV dyssynchrony did not correlate with outcomes. A multimodality approach was effective in predicting the risk for adverse events.

**WHAT IS ALREADY KNOWN ON THIS TOPIC**

- Residual sequelae after surgical repair of tetralogy of Fallot (TOF) can impact long-term outcomes.
- The impact of the ventricular dyssynchrony on biventricular function, exercise capacity and clinical outcome is unknown.

**WHAT THIS STUDY ADDS**

- This study investigated the role of right ventricular dyssynchrony on the long-term prognosis in repair of TOF patients.
- Multimodality approach might help to stratify the risk of outcomes in this population.

**HOW THIS STUDY MIGHT AFFECT RESEARCH, PRACTICE OR POLICY**

- The stratification might help to identify patients with better prognosis as well as high-risk patients, which may require earlier follow-up.

## Introduction

Tetralogy of Fallot (TOF) is the most common cyanotic congenital heart defect (CHD) and is present in 7%–10% of children with CHD.^1^ Since surgical repair was introduced in 1955, long-term prognosis of patients with TOF has improved steadily. Today, more than 95% of children with repaired TOF (rTOF) reach adulthood, compared with less than 20% in historical cohorts without repair.^2^ However, residual sequelae after surgical repair (eg, atrial or ventricular scars, pulmonary valve or right ventricular (RV) outflow tract dysfunction) can impact long-term outcomes by affecting ventricular size and function, and predisposing patients to arrhythmias, sudden cardiac death and heart failure.^3^ So far, most attention has been directed to the negative consequences of residual pulmonary regurgitation on RV size and function and the occurrence of arrhythmias.^4^ Less attention has been paid on the consequences of right bundle branch block and intraventricular dyssynchrony on biventricular function, exercise capacity and clinical outcome.

Echocardiographic 2D-speckle tracking strain analysis allows the assessment of the electromechanical dyssynchrony within the left and right ventricle. Previous studies have shown the prognostic importance of RV myocardial strain for various conditions, such as in patients with idiopathic pulmonary hypertension,^5^ arrhythmogenic RV dysplasia^6^ or with a systemic right ventricle.^7^ With only a few studies performed in children with rTOF,^8^ the prognostic value of RV strain and dyssynchrony in adults with rTOF is still poorly understood. The aim of this study was to investigate the role of RV dyssynchrony surrogates and the risk stratification of patients with rTOF years after surgical repair with clinical parameters.

## Methods

### Study population and characteristics

Adults with rTOF from the multicentre Swiss Adult Congenital HEart disease Registry (SACHER)^9^ followed at the University Hospitals of Bern and Zürich were included. The registry aim was to establish a prospective database of clinical information in order to improve the knowledge base of outcomes in adults with CHD. The inclusion criteria for the actual study were: patients with a Fallot-like physiology such as pulmonary atresia and ventricular septum defect, double outlet RV of Fallot type and complete atrioventricular septal defect with pulmonary stenosis were also included. We defined as baseline visit the first clinical visit between 2012 and 2016, where cardiovascular MR (CMR) was performed, as well as a transthoracic echocardiography (TTE) study was done within a 1-year interval.

Baseline characteristics including age at baseline visit, age at repair, type of repair, gender, body mass index, New York Heart Association functional classification and N-terminal pro–B-type natriuretic peptide (NT-proBNP) levels were derived from the patients’ chart. A 12-lead ECG was part of every clinical visit. The underlying rhythm and QRS durations at baseline were documented. From the cardiopulmonary exercise testing reports the following variables were collected: peak oxygen uptake (pVO_2_), peak heart rate and ventilation for the carbon dioxide production (VE/VCO_2_ slope). Data regarding dimension and function of the heart chambers and severity of tricuspid and pulmonary regurgitation were retrieved from the TTE and CMR charts. All data had to be collected within 6 months before or after the baseline visit.

### Strain analysis and quantification of dyssynchrony

**Figure 1:**
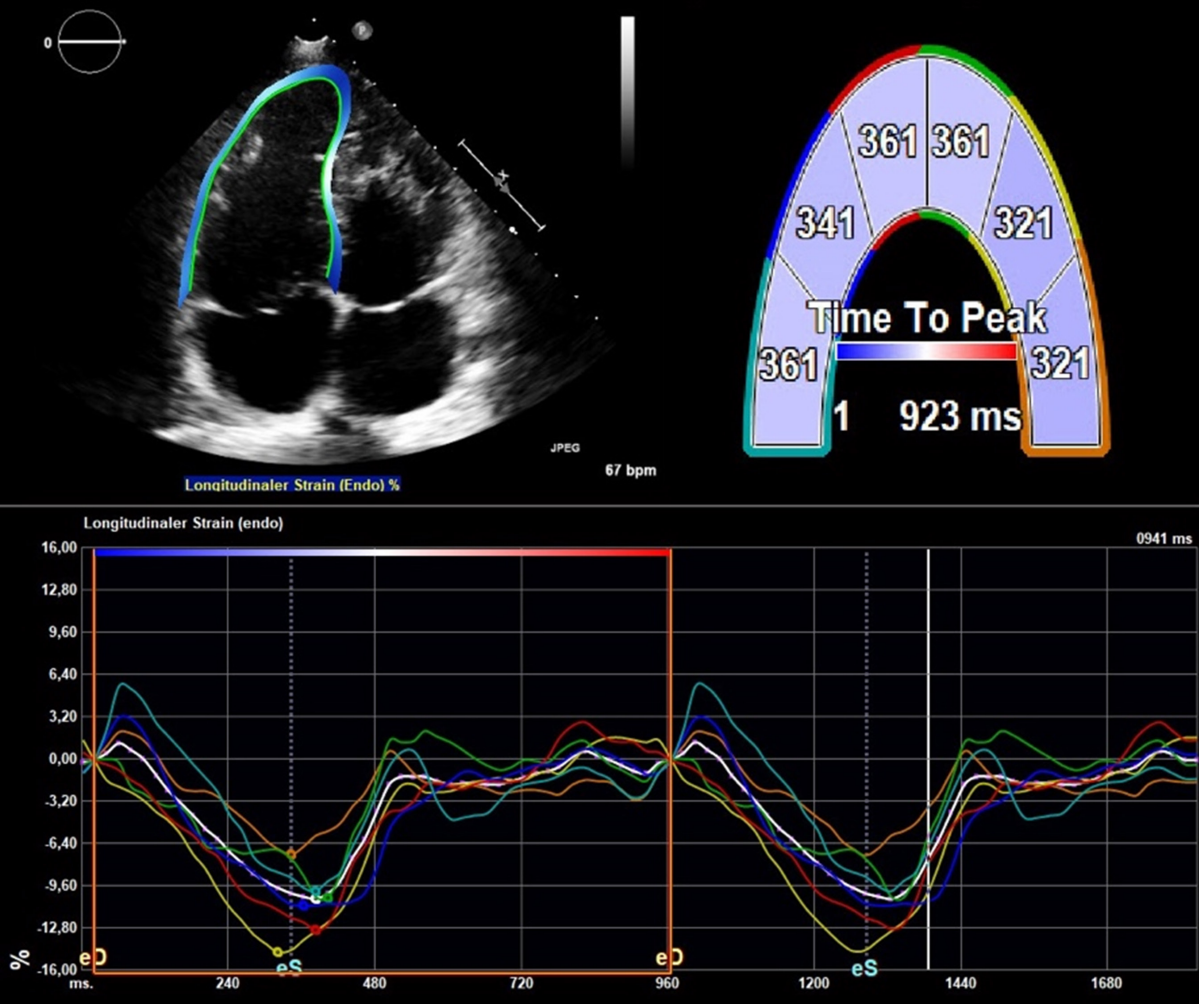
Strain analysis of the right ventricle with measurement of time-to-peak shortening in six right ventricular segments.

2D-Speckle tracking strain analysis was performed offline by one author (AP) using a dedicated software TomTec-Arena V.2.30 (TomTech Imaging Systems, Munich, Germany). Grey-scale images were previously acquired with optimised gain and contrast at frame rates of 50–90 frames/s. RV longitudinal strain was measured in the right ventricle focused four-chamber view. Considering the thin RV wall, measurements were limited to the endocardial strain. The endocardial borders of the right and left ventricle were traced manually excluding papillary muscles and trabeculations. The software-generated tracking was further manually adjusted. Endocardial tracking was accepted for analysis if adequate at visual inspection during the entire cardiac cycle. In case the endocardial border of a wall segment was not evaluable, this echocardiographic examination was excluded from the strain analysis. Strain curves were generated at the basal, mid and apical segments of the RV lateral wall and of the interventricular septum (see figure 1). For each of the six RV segments the time-to-peak myocardial shortening (TTP) was measured. According to Hui *et al*,^10^ maximal RV delay was defined as the difference between the longest and the shortest TTP segment, and the standard deviation (SD) of all segments defined the RV Dyssynchrony Index (RVDI). The median value of RVDI was used to classify the study population into patients with low and high dyssynchrony. Moreover, the strain of the three anterolateral left ventricular (LV) segments was analysed in the apical four-chamber view and interventricular shortening delay (IVSD) was calculated as the maximal TTP difference between any RV and LV lateral walls. Finally, the interventricular ejection delay was defined as the difference between the RV and LV pre-ejection time measured with pulsed-wave Doppler at each ventricular outflow tract. Table 1 summarises the definitions of dyssynchrony surrogates.

**Table 1:** Dyssynchrony variables

TTP	Time-to-peak myocardial shortening: Time interval between the beginning of the QRS complex and peak of systolic shortening.
Max RV delay	Maximal right ventricular delay: Maximal time difference between longest and shortest TTP among the 6 RV segments.
RVDI	RV Dyssynchrony Index: SD of the 6 TTPs.
pET	Pre-ejection time: Time from Q-wave to the beginning of ejection, measured on pw-Doppler at ventricular outflow tract.
IVED	Interventricular ejection delay: Difference between LV-pET and RV-pET.
IVSD	Interventricular shortening delay: Maximal difference between longest and shortest TTP among the RV and LV free wall segments.

LV, left ventricular; SD, standard deviation.

### Clinical outcome

An adverse clinical event was defined as the composite endpoint consisting of all-cause mortality, major arrhythmias and hospital admission for heart failure. Ventricular fibrillation, sustained ventricular tachycardia, atrial flutter and atrial fibrillation requiring cardioversion or ablation therapy were considered major arrhythmias. The time to the first clinical event between baseline visit and 31^st^ December 2019 was analysed.

### Statistical analyses

Continuous variables were reported as mean and SD if normally distributed and compared using Student’s t-test or Welch’s t-test, depending on the equality of the variances. Non-normally distributed variables were reported as median with IQR and compared using the Mann-Whitney U test. Categorical variables were summarised as frequency or proportion and compared using χ^2^ or Fisher’s exact tests. Univariate or multivariate Cox proportional hazard survival models were used to investigate the association between variables of interest and clinical outcome. Harrel’s C-index, also known as concordance index, was calculated as a measure of goodness of fit for every univariate variable. In a second step, a multivariable model consisting of variables with the highest C-index among clinical characteristics, NT-proBNP levels, ECG, imaging or exercise testing data was constructed. The proportional hazards assumption for this model was tested by assessing the relationship between scaled Schoenfeld residuals and time to event. Significant independent predictors of an adverse clinical outcome were used to create a risk score, with the median values of each predictor as cut-off for a score 0 or 1.

To assess intraobserver variability, the same observer (AP) re-analysed strain parameters of 20 randomly selected subjects at the end of the data sampling. For interobserver variability, a second blinded observer (CN) analysed the strain of 20 randomly selected subjects. The interclass correlation coefficient (ICC) using a two-way random/absolute agreement model was used to assess interrater reliability, where a value >0.8 suggests an excellent agreement and value<0.4 indicates poor agreement. A p<0.05 was considered as statistically significant. All data were analysed using STATA software (V.15.1; Stata).

## Results

### Study population

Among the 285 patients, 44% were female with a mean age at baseline 34±14 years. Most of the patients were asymptomatic and in sinus rhythm at baseline visit. The number of patients with a diagnosis of TOF was 244 (86%). A total of 186 patients (65%) underwent a palliation procedure prior to definitive repair. Table 2 summarises the baseline characteristics. Further characteristics are shown in online supplemental table 1.

10.1136/openhrt-2023-002583.supp1 Supplementary data

**Table 2:** Baseline characteristics

Clinical characteristics	Total cohort N=285	No of observations
Age at baseline visit—years	34±14	285
Age at repair—years	6 (1–6)	285
Type of repair		
Non transannular patch	118 (40%)	
Transannular patch	110 (37%)	
RV-PA conduit	56 (19%)	
Unknown	10 (3%)	
Female	126 (44%)	
BMI—kg/m^2^	24.2±4.8	
NYHA functional class		
I	229 (80%)	
II or III	56 (20%)	
NT-proBNP—pg/mL	133 (66–262)	217
Sinus rhythm	259 (91%)	
Atrial fibrillation or flutter	10 (4%)	
Pacemaker rhythm	12 (4%)	
QRS duration—ms	143±26	281
Echocardiography data		
LVEDD—mm	47±7	275
LVESD—mm	32±7	273
LAVI—mL/m^2^	24±15	280
RAA—cm^2^	19±7	247
LVEF—%	59±8	280
RV FAC—%	39±8	272
RV-RA gradient—mm Hg	31±12	254
Tricuspid regurgitation>mild	260 (91%)	275
Pulmonary regurgitation<severe	226 (79%)	273
Strain and dyssynchrony		
RV GLS—%	−15.4±5	222
RV Dyssynchrony Index—ms	40 (28–52)	222
Max RV Delay—ms	95 (62–127)	222
IVED—ms	−3±19	260
IVSD—ms	105±52	209
CMR data		
LV EDVI—mL	88±21	185
RV EDVI—mL	126±35	201
RVEF—%	48±9	208
LVEF—%	57±8	206
Exercise data		
Peak heart rate—bpm	158±27	235
Peak VO_2_—mL/kg/min	22±9	237
VE/VCO_2_ slope	27±5	232

Data are expressed as N (% of total), mean±SD or median (IQR) unless otherwise specified. The number of observations refers to the available data in the registry.

BMI, body mass index; CMR, cardiac MR; EDV, end-diastolic volume; EDVI, end-diastolic volume indexed; GLS, global longitudinal strain; IVED, interventricular ejection delay; IVSD, interventricular shortening delay; LAVI, left atrial volume indexed; LVEDD, left ventricular end-diastolic diameter; LVEF, left ventricular ejection fraction; LVESD, left ventricular end-systolic diameter; NT-proBNP, N-terminal pro–B-type natriuretic peptide; NYHA, New York Heart Association; RAA, right atrial area; RVEF, right ventricular ejection fraction; RV FAC, RV fractional area change; VE/VCO_2_, minute ventilation/carbon dioxide output; VO_2_, oxygen uptake.

### RV dyssynchrony

In 222 patients (78%), echocardiographic imaging quality was sufficient to measure the TTP in all 6 RV segments. On average, the median of maximal RV delay was 95 ms (62–127 ms) and the median RVDI was 40 ms (28–52 ms). Patients with above-median RVDI had a larger left atrium and ventricles, lower LV ejection fraction and lower RV global longitudinal strain (GLS). The proportion of patients with severe pulmonary regurgitation was higher in the group higher RV dyssynchrony (15% vs 25%, p=0.023). No significant differences were observed in NT-proBNP levels, cardiopulmonary exercise variables or outcomes (see table 3).

**Table 3:** Comparison between patients with higher and lower RV Dyssynchrony Index (RVDI)

	Patients with RVDI<median N=113	Patients with RVDI≥median N=109	P value
Clinical characteristics			
Age at repair—years	5.7 (1–9)	4.7 (1–6)	0.35
Age at baseline—years	33±12	34±13	0.45
Primary repair	73 (65%)	71 (65%)	0.9
NYHA functional class I	94 (83%)	93 (85%)	0.8
NT-proBNP—pg/mL	119 (57–983)	153 (70–1666)	0.3
No device (ICD, PM, CRT)	104 (92%)	98 (89%)	0.7
QRS duration—ms	143±25	144±27	0.91
Adverse clinical events	10 (9%)	14 (13%)	0.3
Echocardiography data			
LVEDD—mm	46±6	48±7	0.008
LVESD—mm	30±5	33±7	0.001
LVEF—%	60±8	58±8	0.05
LAVI—mL/m^2^	24±10	29±17	0.04
RA area—cm^2^	19±7	20±7	0.12
RV-RA gradient—mm Hg	32±12	30±12	0.21
Severe tricuspid regurgitation	2 (1.8%)	1 (0.6%)	0.147
Severe pulmonary regurgitation	16 (14.5%)	43 (24.5%)	0.023
RV FAC—%	38±7	39±9	0.4
RV GLS—%	−16±4.4	−14.6±5	0.032
IVSD—ms	86±38	126±56	<0.001
IVED—ms	−4.5±15.9	−1.6±21.9	0.3
CMR data			
LV EDVI—mL	83±17	90±23	0.007
RV EDVI—mL	122±28	137±19	0.004
RV EF—%	47±8	47±8	0.8
LV EF—%	60±8	57±9	0.02
Exercise data			
Peak VO_2_—mL/kg/min	21±9	21±9	0.9
Peak heart rate—bpm	159±30	158±26	0.7
VE/VCO_2_	26±5	27±5	0.6

Data are expressed as N (% of total), mean±SD or median (IQR) unless otherwise specified.

CMR, cardiac MR; CRT, cardiac resynchronization therapy; EDVI, end-diastolic volume indexed; FAC, fractional area change; GLS, global longitudinal strain; ICD, Implantable cardioverter-defibrillator; IVED, interventricular ejection delay; IVSD, interventricular shortening delay; LAVI, left atrial volume indexed; LVEDD, left ventricular end-diastolic diameter; LVEF, left ventricular ejection fraction; LVESD, left ventricular end-systolic diameter; NYHA, New York Heart Association; PM, pacemaker; RV EDV, right ventricular end-diastolic volume; RVEF, right ventricular ejection fraction; VE/VCO_2_, minute ventilation/carbon dioxide output; VO_2_, oxygen uptake.

### Clinical outcomes

During a mean follow-up of 48±21 months, 33 patients (12%) suffered an adverse clinical outcome. Overall, 13 (4.5%) patients suffered an atrial flutter or fibrillation, while 11 (3.9%) patients were hospitalised due to decompensated heart failure. Five patients (1.8%) experienced a ventricular arrhythmia and 4 patients (1.4%) died during the follow-up. Patients who experienced an adverse event during follow-up were older, had undergone repair later in life and had more often a palliative procedure prior to complete cardiac repair. These patients were more symptomatic, had higher NT-proBNP levels and longer QRS duration. In the event group the left ventricle and both atria were larger, the RV fractional area change was lower and the RV systolic pressure higher. The proportion of patients with severe tricuspid or pulmonary regurgitation was significantly higher in the event group. Their exercise capacity, peak heart rate and the ventilatory efficiency were lower compared with patients without event. No differences were found with respect to surrogate markers of RV dyssynchrony and myocardial deformation. Patient characteristics and clinical findings stratified by events are shown in table 4. The type of events and further details are summarised in online supplemental table 2.

**Table 4:** Comparison of the baseline characteristics between patients with and without event

	Patients without event N=252 (88%)	Patients with event N=33 (12%)	P value
Clinical characteristics			
Age at repair—years	2.6 (2.2–35.6)	6.2 (2.2–29.9)	0.023
Age at baseline—years	29.3 (22.2–65.8)	47.5 (33.1–67.5)	<0.001
Primary repair	172 (68)	14 (42)	0.003
NYHA functional class I	210 (83)	19 (58)	0.001
NT-proBNP—pg/mL	120 (59–983)	434 (191–1871)	<0.001
No device (ICD, PM, CRT)	236 (94)	27 (82)	0.011
QRS duration—ms	142±25	156±27	0.004
Echocardiography data			
LVEDD—mm	46±6	50±8	0.016
LVESD—mm	31±6	35±9	0.012
LVEF—%	59±8	57±11	0.31
LAVI—mL/m^2^	24±11	38±21	0.004
RA area—cm^2^	18±6	25±6	<0.001
RV-RA gradient—mm Hg	30±12	39±10	<0.001
RV FAC—%	39±8	35±11	0.03
Severe tricuspid regurgitation	1 (0.4%)	2 (6.3%)	0.002
Severe pulmonary regurgitation	47 (18%)	12 (36%)	0.014
RV GLS—%	−16±5	−15±6	0.38
RV Dyssynchrony Index—ms	49±31	41±18	0.21
IVSD—ms	103±50	130±65	0.09
IVED—ms	−3.3±19	2.5±19	0.12
CMR data			
LV EDVI—mL	86±19	99±31	0.99
RV EDVI—mL	124±31	139±56	0.95
RV EF—%	43±13	48±8	0.11
LV EF—%	57±7	53±11	0.02
Exercise data			
Peak VO_2_—mL/kg/min	22±9	18±11	0.03
Peak heart rate—bpm	161±24	133±33	0.003
VE/VCO_2_	26±5	30±5	<0.001

Data are expressed as N (% of total), mean±SD or median (IQR) unless otherwise specified.

CMR, cardiac MR; CRT, cardiac resynchronization therapy; EDVI, end-diastolic volume indexed; RV FAC, right ventricular fractional area change; GLS, global longitudinal strain; ICD, Implantable cardioverter-defibrillator; IVED, interventricular ejection delay; IVSD, interventricular shortening delay; LAVI, left atrial volume indexed; LVEDD, left ventricular end-diastolic diameter; LVEF, left ventricular ejection fraction; LVESD, left ventricular end-systolic diameter; NT-proBNP, N-terminal pro–B-type natriuretic peptide; NYHA, New York Heart Association; PM, pacemaker; RV EDV, right ventricular end-diastolic volume; RVEF, right ventricular ejection fraction; VE/VCO_2_, minute ventilation/carbon dioxide output; VO_2_, oxygen uptake.

### Univariate and multivariate predictors of events

By univariate Cox regression analysis, age at repair and at baseline visit, NYHA class, NT-proBNP levels and QRS duration were significantly associated with adverse clinical events. Age at inclusion and NT-proBNP showed the highest association. Among all echo and CMR imaging data, the dimensions of left and right atrial area (RAA) showed the highest association with outcome and exceeded markers of LV and RV size and function, as well as all surrogates of ventricular dyssynchrony. Using a multivariate Cox-regression model, NT-proBNP, RAA and peak heart rate were independent predictors of outcomes after correction for age at baseline. Online supplemental table 3 and online supplemental figure 1 provide detailed results of the univariate and multivariate analysis.

10.1136/openhrt-2023-002583.supp2 Supplementary data

**Figure 2:**
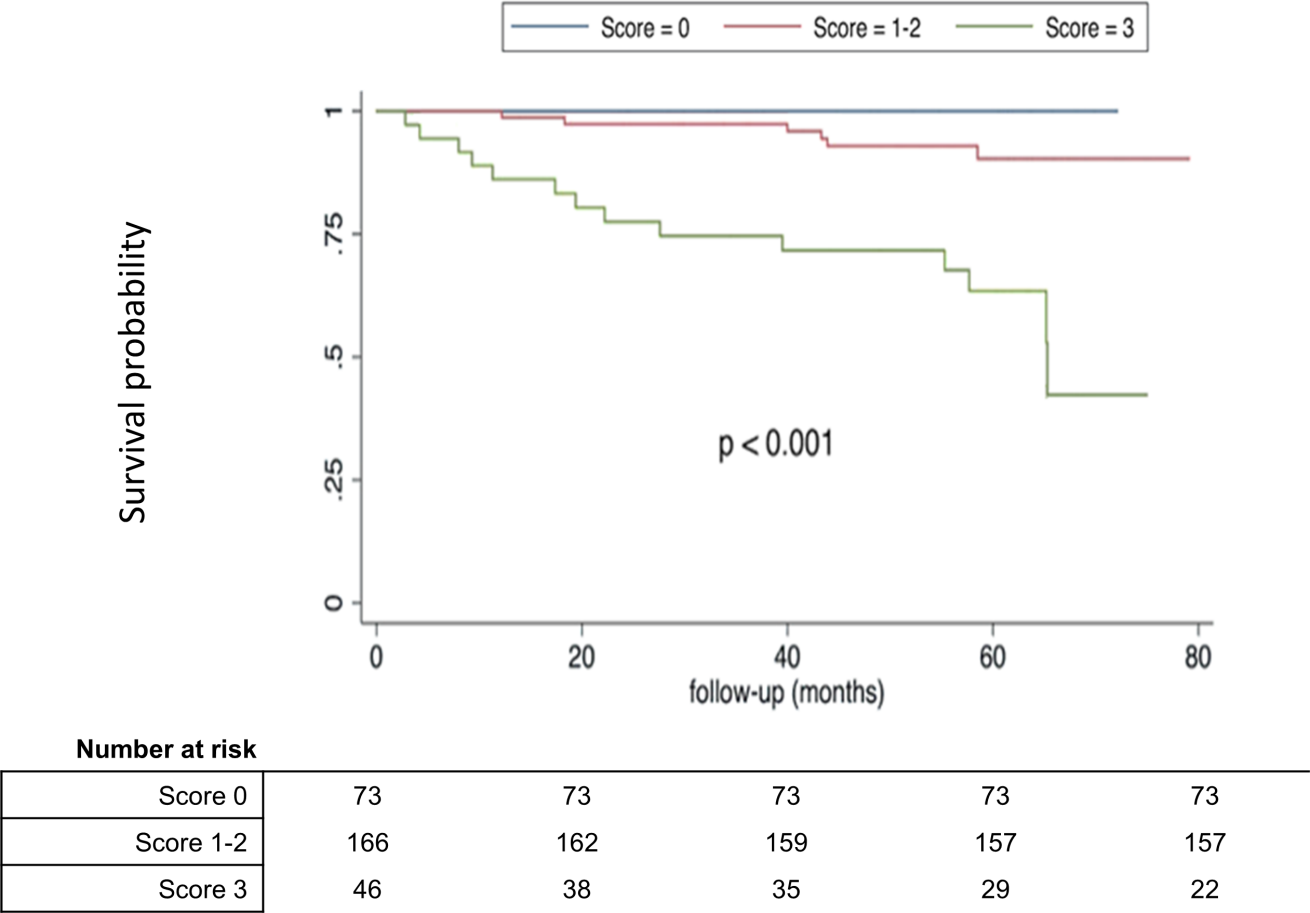
Kaplan-Maier event-free survival curve according to the risk score of 0, 1–2 and 3. 1 point is given if NT-proBNP>133 pg/mL, 1 point if right atrial area >18 cm^2^ and 1 point if peak heart rate <164 bpm. NT-proBNP, N-terminal pro–B-type natriuretic peptide.

Using the median values of the variables as cut-off (NT-proBNP>133 pg/mL, RAA>18 cm^2^ and peak heart rate<164 bpm), a risk score was calculated. Patients with NT-proBNP values >133 pg/mL had a 5.9-fold increased risk (95% CI 2.1 to 17.1) of an adverse event compared with patients with values below median. Patients with RAA>18 cm^2^ and a peak heart rate <164 bpm had an HR of 5.1 (95% CI 1.9 to 13.5) and 5.3 (95% CI 1.8 to 15.3), respectively. The combined score accurately stratified patients into low-risk (score 0; n=39) and high-risk (score 3; n=38) for an adverse event, as depicted in the Kaplan-Meier curve (figure 2 and online supplemental figure 2). After a mean follow-up of 4 years, none of the patients with a score of 0 had an adverse event, while 62% of patients with score of 3 experienced adverse events (p<0.001).

### Intraobserver and interobserver variability

According to the ICC, the intraobserver and interobserver variability was 0.90 and 0.55 for RV GLS and 0.83 and 0.50 for the RVDI, respectively. Mean difference and SD for intraobserver comparison was 0.6±2 and – 2.8±3.0 for RV GLS and for RVDI, respectively. Similarly, mean difference and SD for interobserver comparison was −1.6±3 and −0.5±16 for RV GLS and for RVDI, respectively.

## Discussion

The aim of this study was to investigate the role of RV dyssynchrony surrogates and the risk stratification of patients with rTOF years after surgical repair with clinical parameters. The main finding was that markers of RV dyssynchrony did not predict clinical events over a mean follow-up of 4 years. However, a multimodality approach including NT-proBNP levels, RAA and peak heart rate during exercise testing was very useful in identifying patients at low and high risk of an adverse event.

### RV dyssynchrony

Almost all adults with rTOF have a right bundle branch block on ECG, as a result of either right ventriculotomy, infundibular muscular resection, patch closure of the ventricular septal defect or due to a combination of injuries to the right bundle branch.^11^ Delayed electrical activation of RV segments contributes to electromechanical dyssynchrony. Other factors, such as RV regional wall stress, thickness and shape can also affect RV mechanical dispersion.^12^ At present, a single widely accepted definition of RV dyssynchrony is still lacking.^13^ In this study, we focused on RVDI as defined by Hui *et al*,^10^ taking into account all six RV segments. In our cohort, we found no correlation between ventricular mechanical dispersion and clinical outcomes. Similar results have been reported by other groups using CMR to identify RV dyssynchrony. For instance, Moon *et al*^14^ showed that LV and RV CMR-derived strain parameters of ventricular function were associated with death or ventricular tachycardia in adults with rTOF, but no such correlation was found for ventricular dyssynchrony. Jing *et al*^15^ reported that CMR-derived RVDI was not predictive of changes in RV size and function over time in a large cohort of adults with rTOF. In a study with children after TOF-repair, RVDI by echocardiography was found to have a weak correlation with RV remodelling and function by univariate analysis.^8^ In this study, RV septal delay and prestretch duration were independent predictors of outcomes. These results suggest that RV dyssynchrony, in contrast to RV strain measures, is less associated with adverse clinical outcomes. This is in contrast with other conditions such as idiopathic pulmonary hypertension or in patients with systemic RV, where the predictive value of RV dyssynchrony has been well described.^16 17^ One could conclude that mechanical dispersion caused by surgical scars after repair might be less related to outcome than the RV mechanical dispersion due to a pressure-overloaded. This is supported by the fact that, until now, no prognostic risk scores proposed in the literature for rTOF employ dyssynchrony parameters.^18^ Further studies are needed to validate these hypotheses.

### Multimodality risk stratification

Our study demonstrates that risk stratification with NT-proBNP levels, RAA and peak heart rate is highly predictive for identifying low-risk and high-risk patients for adverse event. Elevated NT-proBNP levels are well known to predict adverse events in patients with CHD.^19^ In our study, NT-proBNP levels of 133 pg/mL predicted the composite outcome of death, decompensated heart failure and major arrhythmias (AUC 0.83). These results are comparable with those reported by Westhoff-Bleck *et al*^20^ in a cohort of rTOF patients, where NT-proBNP levels of 126 pg/mL predicted a composite outcome with an AUC of 0.87.

A dilated right atrium is related to supraventricular arrhythmia in TOF patients,^21^ as the atrial stretch alters the electrical refractoriness enhancing the intrinsic propensity to reentry.^22^ In our analysis, an RAA>18 cm^2^ was a significant predictor of adverse outcomes. Diller *et al*^23^ showed that a RAA>22 cm² on CMR identified rTOF patients at increased risk of death or malignant arrhythmias during follow-up. Of note, the cut-off proposed in our study (RAA>18 cm^2^) is identical to the one used in patients with pulmonary arterial hypertension to assess their risk of adverse events.^24^

In addition to neurohormones and imaging data at rest, cardiopulmonary exercise testing variables reflect the patients’ haemodynamic reserve at exercise. In adults with rTOF, a correlation between mortality and peak heart rate, pVO_2_ and the VE/VCO_2_ slope has been demonstrated.^25^ In our cohort, these three variables were all predictors of a clinical adverse events at univariate analysis. However, in the multivariate Cox regression only peak heart rate remained an independent predictor. Peak heart rate is easily and reliably measurable and appears to be a crucial parameter of exercise performance in rTOF.^26^ Patients with surgically repaired CHD often experience chronotropic incompetence, resulting in reduced maximal heart rate and compromised exercise performance.^27^ The mechanisms underlying the diminished chronotropic response in rTOF remain inadequately understood. Although therapy with beta-blockers may also play a role, recent studies suggest that autonomic dysfunction, neurohormonal activation and cardiac arrhythmias may also be associated with chronotropic incompetence in the adult CHD populations.^28^ Overall, decreased peak heart rate may be a surrogate of advanced heart disease, and not simply the effect of beta-blockers.

### Composite score for risk prediction

Combining three established predictors yielded an easily applicable risk stratification tool that was very useful to identify patients at low risk (score 0) and high risk (score 3) of adverse events. In our study, these two groups represented half of our cohort (25%, 25%). A score of 0 (no risk factor present) reliably identified patients at low risk, whereas a score of 3 (all risk factors present) was highly specific in identifying patients at risk of an adverse event. This reflects the independence of all three risk factors.

### Study limitations

Our study has several limitations. First, this was a retrospective study with limitations related to such a design. Second, the number of patients who experienced adverse events was small, limiting the statistical power of the study. Third, not all measures could be obtained in all patients, further reducing the number of observations. Furthermore, despite growing evidence of the prognostic role of late-gadolinium-enhancement in CMR in rTOF patients, this feature was not investigated in the routine CMR of our cohort.

Another potential limitation of our study relates to the method of assessing RV longitudinal strain. We measured the strain of both the RV free wall and the septal segments for dyssynchrony analysis. There is an ongoing debate about whether to include the right side of the interventricular septum in the assessment of RV longitudinal strain or not. Current recommendations of the American Society of Echocardiography and the European Association of Cardiovascular Imaging do not provide clear guidance.^29^ Excluding the interventricular septum from RV strain analysis might have a superior prognostic value in some cardiovascular diseases, nonetheless, it would limit the ability to assess mechanical dispersion within the right ventricle.

Finally, we found only a moderate interobserver reproducibility of the RVDI, which is consistent with the findings of the EACVI-ASE Strain Standardisation Task Force.^30^ This may have implications for the application of RVDI in clinical practice, as it suggests that RVDI measurement may be operator-dependent.

## Conclusion

In our study, we found no correlation between RV dyssynchrony markers and the occurrence of adverse events during a 4-year follow-up period in adults with rTOF. A risk score combining NT-proBNP levels, RAA and peak heart rate was highly predictive in stratifying patients at low and high risk. Further prospective validation studies are required to confirm the prognostic value of this score.

## Data Availability

No data are available.
