# Supplementary material for: Prognostic value of right ventricular dyssynchrony in adults with repaired tetralogy of Fallot
Source: Open Heart. 2024 Jan 19;11(1):e002583. doi: 10.1136/openhrt-2023-002583 (PMC10806502; doi:10.1136/openhrt-2023-002583)
Supplement: Supplementary data [file openhrt-2023-002583supp001.pdf]

SUPPLEMENTARY TABLES

Supplementary Table 1 – Baseline characteristics	
**Diagnosis**	
Tetralogy of Fallot	244 (86%)
Double outlet RV	34 (12%)
Pulmonary atresia with VSD	4 (1%)
Complete AVSD with PS	3 (1%)
**Interventions**	
Palliation before repair	186 (65%)
Direct repair	99 (35%)
Type of repair	
Transannular patch	132 (46%)
No transannular patch	116 (41%)
RV-PA-Conduit placement	29 (10%)
Unknown	8 (3%)
Number of reinterventions	
0	112 (39%)
1	103 (36%)
2	38 (13%)
3	20 (7%)
4 or more	12 (4%)
**Residual lesions**	
Peak RVOT gradient – mmHg	23 ± 14
RV-RA gradient – mmHg	31 ± 12
Pulmonary insufficiency	279
None to mild	42 (15%)
Moderate	178 (64%)
Severe	59 (21%)

Data are expressed as N (% of total) or mean ± standard deviation

Supplementary Table 2 – Outcomes	Total cohort N = 285
Age at last follow-up – years	35 [27 - 47]
Time to follow-up – months	48 ± 21
Time to event – months	30 ± 21
Adverse clinical event	33 (11.6%)
- All-cause mortality	4 (1.4%)
- Clinically relevant arrhythmia	
o AFib/AFl	13 (4.5%)
o VT/VF	5 (1.8%)
- Heart failure	11 (3.9%)

Data are expressed as N (% of total) or mean ± standard deviation.
Abbreviations : AFib, atrial fibrillation ; AFl, atrial flutter ;
VF, ventricular fibrillation ; VT, ventricular tachycardia.

Supplementary Table 3				
**Univariate Cox-Regression model**				
**Clinical characteristics**	**HR**	**95% CI**	**p-value**	**C index**
Age at repair (years)	1.05	1.02-1.08	<0.001	0.69
Age at baseline (per 10 years)	1.70	1.40-2.05	<0.001	0.76
Palliation prior to repair	2.12	1.08-5.34	0.30	0.61
NYHA > I	3.6	1.8-7.2	<0.001	0.65
NT-proBNP (per 1000 pg/ml)	1.20	1.05-1.37	0.006	0.80
QRS duration (per 10 ms, N=273)	1.24	1.07-1.44	0.005	0.62
**Echocardiography Data**				
LVEDD	1.09	1.03-1.15	0.002	0.63
LVEF	0.97	0.93-1.01	0.19	0.64
LAVI	1.04	1.02-1.06	<0.001	0.73
RAA	1.08	1.04-1.11	<0.001	0.76
RV-RA gradient (per 10 mmHg)	1.49	1.17-1.89	0.001	0.71
RV FAC	0.94	0.90-0.98	0.007	0.59
RV GLS	0.97	0.88-1.07	0.54	0.58
RV Dyssynchrony Index	1.02	0.99-1.03	0.11	0.55
Severe tricuspid regurgitation	1.05	0.42- 2.58	0.923	0.52
Severe pulmonary regurgitation	2.05	1.14-3.67	0.02	0.63
IVSD	1.01	1.00-1.01	0.03	0.65
IVED	1.01	0.99-1.03	0.18	0.52
**CMR Data**				
LV EDVI	1.01	0.99-1.01	0.06	0.50
RV EDVI	1.01	1.00-1.01	0.02	0.58
RV EF	0.95	0.92-0.99	0.01	0.56
LV EF	0.94	0.90-0.99	0.01	0.56
**Exercise data**				
pVO_2	0.94	0.9-0.99	0.04	0.65
Peak heart rate	0.97	0.96-0.98	<0.001	0.75
VE/VCO_2	1.11	1.05-1.16	<0.001	0.71
**Multivariate Cox- Regression analysis**				
Age at baseline (per 10 years)	1.10	0.76-1.57	0.13	
NT-proBNP (per 1000 pg/ml)	1.37	1.01-1.77	0.013	
RA area (cm^2)	1.05	1.00-1.10	0.044	
Peak heart rate (bpm)	0.97	0.96 -0.99	0.002	

For abbreviations see Table 2.
