# Supplementary material for: Prognostic value of right ventricular dyssynchrony in adults with repaired tetralogy of Fallot
Source: Open Heart. 2024 Jan 19;11(1):e002583. doi: 10.1136/openhrt-2023-002583 (PMC10806502; doi:10.1136/openhrt-2023-002583)

Supplementary Figure 1

Forest plot of the Cox Proportional Hazards Model

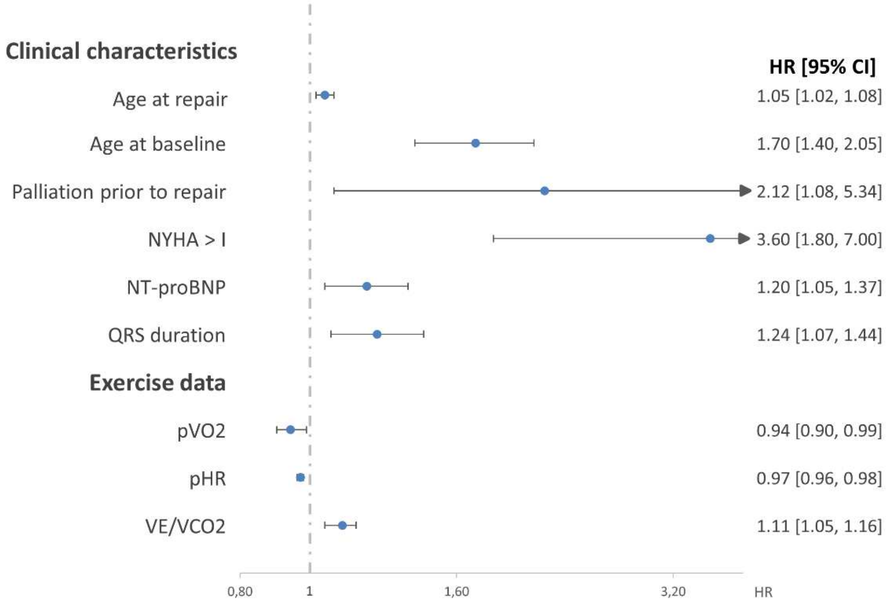

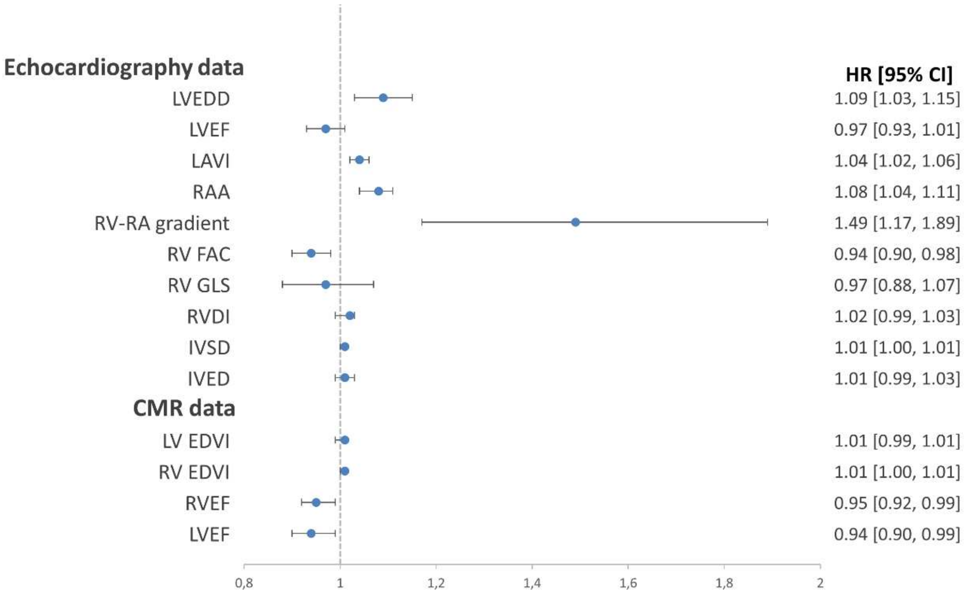

Supplementary Figure 2

Kaplan-Maier event-free survival curve according to median value of NT-proBNP (133 pg/ml), right atrial area (18 cm^2) and peak heart rate (164 bpm)

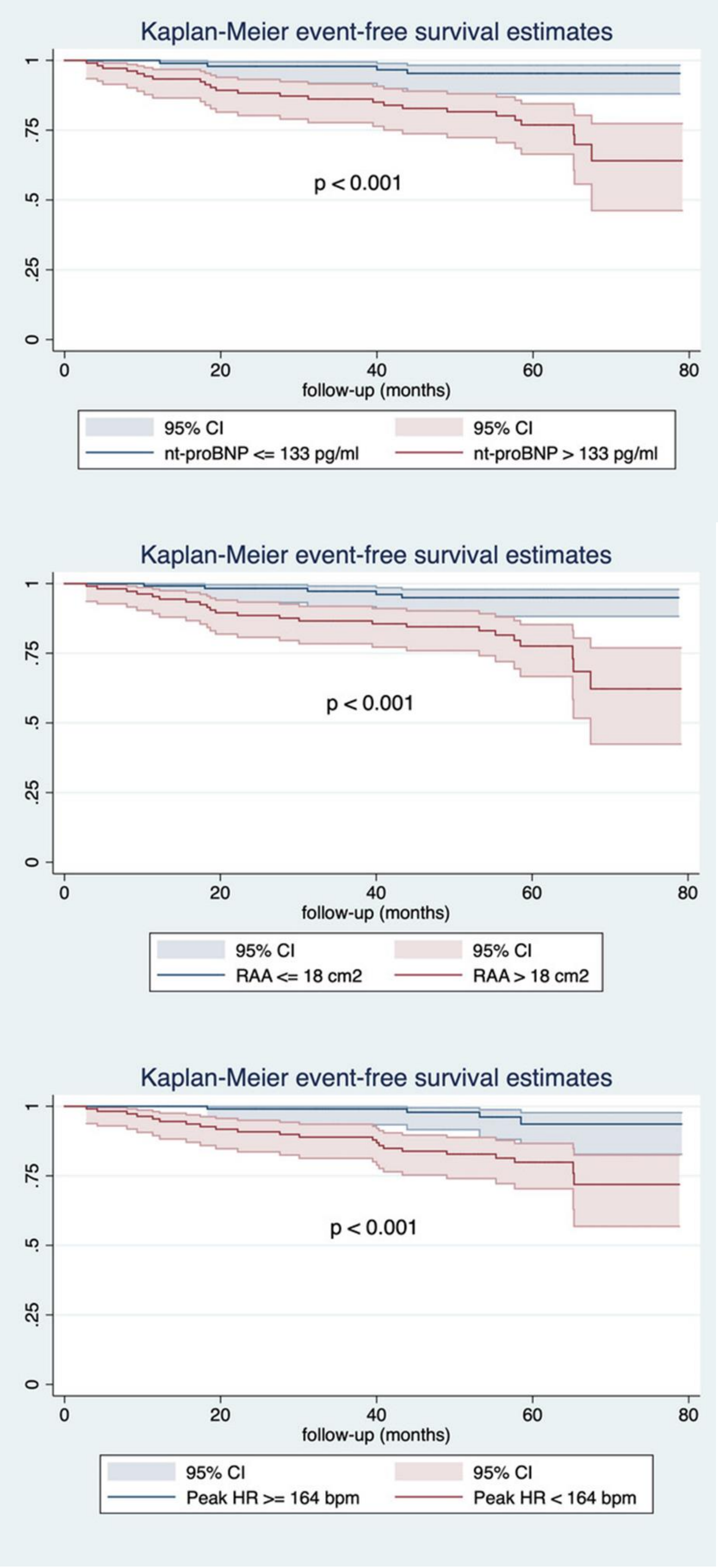

Supplement: Supplementary data [file openhrt-2023-002583supp002.pdf]
